# The burden of pre-extensively and extensively drug-resistant tuberculosis among MDR-TB patients in the Amhara region, Ethiopia

**DOI:** 10.1371/journal.pone.0229040

**Published:** 2020-02-13

**Authors:** Agumas Shibabaw, Baye Gelaw, Wondwossen Gebreyes, Richard Robinson, Shu-Hua Wang, Belay Tessema

**Affiliations:** 1 Department of Medical Microbiology, School of Biomedical and Laboratory Sciences, College of Medicine and Health Sciences, University of Gondar, Gondar, Ethiopia; 2 Global One Health Initiative (GOHi), The Ohio State University, Columbus, Ohio, United States of America; 3 Department of Microbial Infection and Immunity, College of Medicine, The Ohio State University, Columbus, Ohio, United States of America; 4 Department of Veterinary Preventive Medicine, College of Veterinary Medicine, The Ohio State University, Columbus, Ohio, United States of America; 5 Department of Internal Medicine, Division of infectious diseases, College of Medicine, The Ohio State University, Columbus, Ohio, United States of America; Institute of Medical Sciences, Banaras Hindu University, INDIA

## Abstract

**Background:**

The emergence of pre-extensively and extensively drug-resistant tuberculosis (Pre-XDR/XDR-TB) is the major hurdle for TB prevention and care programs especially in developing countries like Ethiopia. The less emphasis on universal access to laboratory techniques for the rapid diagnosis of TB and drug susceptibility testing (DST) makes the management of MDR-TB a challenge. Early detection of second line anti-TB drugs resistance is essential to reduce transmission of Pre-XDR/XDR-TB strains and adjusting the treatment regimen in MDR-TB.

**Objective:**

To determine the prevalence and resistance pattern of Pre-XDR- and XDR-TB among MDR-TB patients in the Amhara region, Ethiopia.

**Methods:**

A cross sectional study was carried out in nine MDR-TB treatment centers in the Amhara region. Sputum samples were collected from all pulmonary rifampicin resistant (RR) or MDR-TB patients prior to anti-TB treatment. Lӧwenstein-Jensen (LJ) culture, Ziehl Neelsen (ZN) smear, MTBDRplus and MTBDRsl assays were performed according to the standard procedures. Data were analyzed using SPSS 20 software. Chi-square and/or Fishers exact test was employed.

**Results:**

Overall, 6.3% of MDR-TB isolates were resistant to at least one second line drugs. Pre-XDR-TB and XDR-TB isolates accounted 5.7% and 0.6% respectively. Moreover, 3.4% were resistant to FQs and 3.4% were resistant to second line injectable drugs. All isolates were susceptible for low level kanamycin. Almost all pre-XDR-TB strains (90%) were previously treated with anti-TB drugs. Drug resistant *Mycobacterium tuberculosis* isolates were disproportionately distributed in districts of the Amhara region and the majorities were concentrated in urban areas.

**Conclusions:**

The high proportion of MDR-TB patients resistant to at least one second line drug is alarming. Strengthening the laboratory facilities to monitor pre-XDR and XDR-TB patients is crucial. The TB programs need to give emphasis on the effective and rational use of second line drugs for newly diagnosed MDR-TB patients to prevent the emergence of pre-XDR/XDR-TB strains.

## Introduction

The emergence of multidrug-resistant tuberculosis (MDR-TB) and extensively drug-resistant tuberculosis (XDR-TB) is a global public health crisis. MDR-TB is defined as TB strains resistant to at least two first line drugs, rifampin (RIF) and isoniazid (INH). Pre-extensively drug resistant TB (Pre-XDR TB) is defined as MDR-TB strain that is resistant to either fluoroquinolones (FQ) or second line injectable drug but not both. XDR-TB is defined as MDR-TB strain that is resistant to any FQs and one of the second line injectable drugs (capreomycin, kanamycin or amikacin) [1].

The latest Global TB Report estimates that 3.4% new and 18% of previously treated persons with TB diagnosed in 2018 were RR/MDR-TB. In 2018, approximately 484,000 people developed RR/MDR-TB globally. Globally, 6.2% MDR-TB cases were estimated to have XDR-TB in 2018 [2]. Ethiopia currently ranks 3^rd^ in Africa in TB incidence and among the 30 high burden countries with high incidence of TB, TB/HIV and MDR-TB. Furthermore, the rate of RR/MDR-TB was 2.7% and 14% among new and previously treated cases respectively, and four XDR-TB cases were reported in Ethiopia [2, 3].

Drug-resistant strains of *Mycobacterium tuberculosis* arise due to spontaneous chromosomal mutations at a low frequency, but one study revealed that selection pressure that is caused by inappropriate utility of anti-TB drugs results in the emergence of drug resistant TB [4]. Resistance to first and second line anti-TB drugs has been linked to mutations of genes: *Kat*G and *inh*A for isoniazid resistance, *rpo*B for rifampicin resistance; *gyr*A and less frequent *gyr*B for FQ resistance; *rrs* and *eis* promoter region for aminoglycosides (amikacin/kanamycin); *rrs* and *tly*A for capreomycin resistance [5, 6].

A person can get drug resistant TB through primary direct transmission and secondary due to inadequate TB treatment for extended duration of time. Pre-XDR and XDR-TB patients are usually treated with regimens that include group V drugs (clofazimine, linezolid, high dose INH, bedaquiline, delamanid) as they are usually resistant to most of the effective drugs and treatment outcome in these patients is poor [7].

FQ is the most effective second line ant-TB drug and recommended for the treatment of MDR-TB [8]. MDR-TB patient treatment is further complicated due to FQ resistance (Pre-XDR-TB), that lead to longer duration of treatment, limited treatment options and results in poor outcome [9]. The early detection of Pre-XDR/XDR-TB could guide clinicians in the appropriate adjustment of MDR-TB treatment regimen with effective drugs to prevent treatment failure.

The geographic distribution of the disease varies worldwide, as well as within countries, due to poverty and other risk factors [10, 11]. Delayed DST before MDR-TB diagnosis could result in further transmission of drug resistant TB strains and use of inappropriate regimen containing SLDs would increase the risk of resistance to SLDs [12].

The global incidence of FQ resistance among RR/MDR-TB cases was 21% according to WHO 2019 TB report [2] but unfortunately, till date limited data are available on prevalence of Pre-XDR-TB worldwide, including Ethiopia. Exposure to second line drugs for the treatment of respiratory tract infections other than TB contribute to evolution of resistance to these drugs and delays diagnosis of TB [13–15].

The use of geographical information system (GIS) [16], is important to show the geographical distribution of strains, locate hotspots of the disease and related ecological factors of drug resistant TB in high TB burden countries like Ethiopia. Therefore, second line drugs are routinely used in the country and it is important to determine the prevalence and resistance pattern of FQs and second line injectable drugs at baseline among MDR-TB isolates and show the distribution of these strains at district level.

## Materials and methods

### Study setting and design

**Fig 1:**
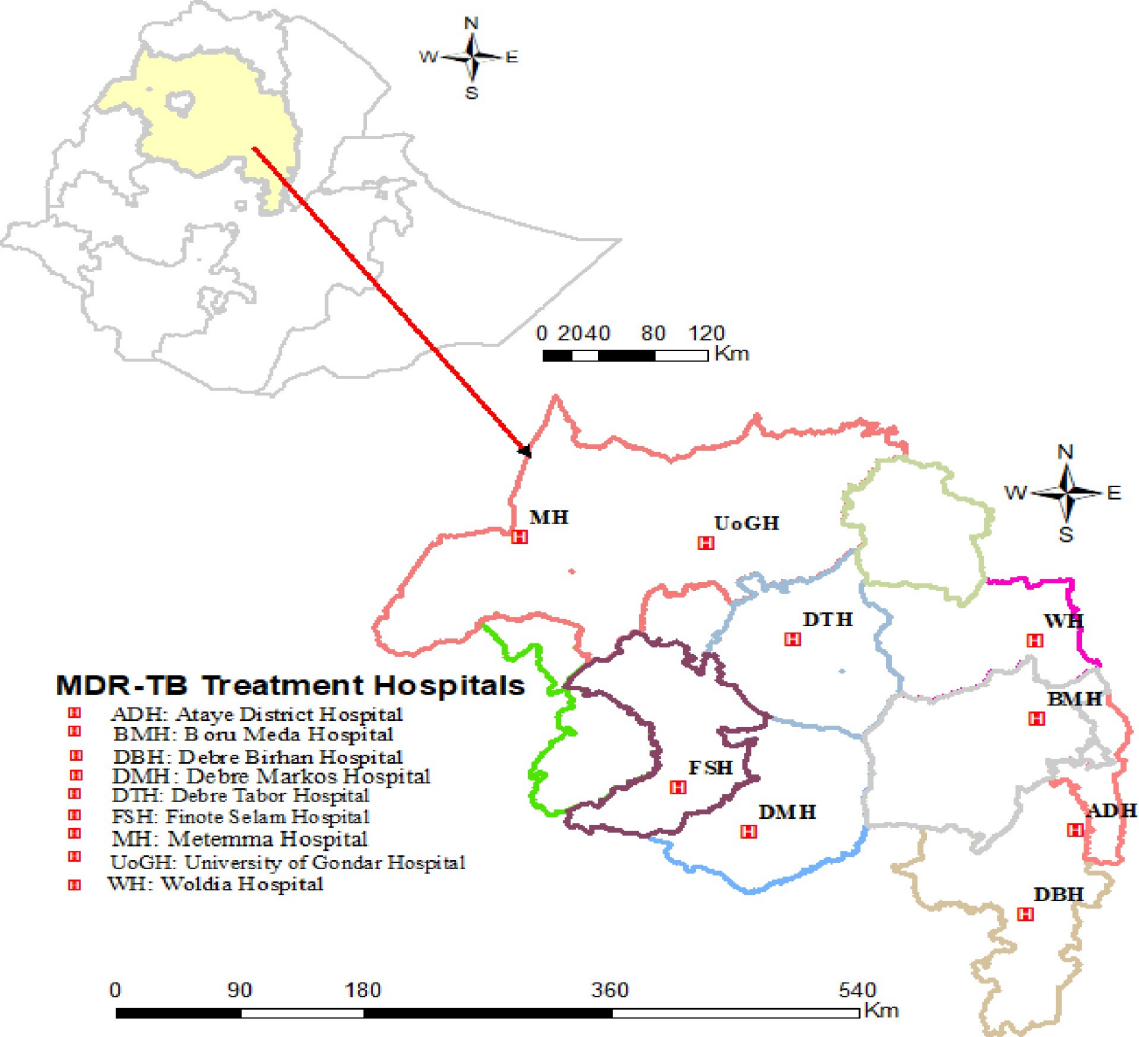
Geographical location of the study area and RR/MDR-TB treatment center hospitals in the Amhara region.

A cross sectional study was conducted from January 2016 to September 2018 at nine MDR-TB treatment center hospitals in the Amhara region (Fig 1).

All pulmonary RR/MDR-TB patients or patients who failed treatment of first line drug (FLD) who were 15 years of age or older and admitted in MDR-TB treatment centers were eligible for enrollment in the study. Extra-pulmonary RR/MDR-TB patients and those pulmonary MDR-TB patients who started second line drugs (SLDs) during data collection were excluded. In Ethiopia, drug resistance TB diagnosis has been carried out using GeneXpert MTB/RIF assay, genotypic and phenotypic drug susceptibility testing (DST). However, these tests are only available at a few referral health facilities or institutions for detection of RR/MDR-TB. Once the patient is diagnosed with RR/MDR-TB or has history of prior TB, they will be referred to the nearby MDR-TB treatment center health facilities for admission and treatment initiation with SLDs. Demographic, clinical and laboratory data including HIV tests were collected. In addition, the zonal, district and kebele of each study participants were registered.

### Specimen collection, processing and laboratory analysis

Five to 10 ml of sputum samples were collected into a sterile 50 ml falcon tube at baseline and all specimens were stored at -20°C until transported to University of Gondar Hospital TB culture (UoGH-TBCL) or Amhara Public Health Institute (APHI) TB laboratory using cold chain. Sputum samples were decontaminated using N-Acetyl L-cysteine- Sodium Hydroxide (NALC-NaOH) solution and neutralizing by phosphate buffer. Centrifuge the mixture and then the sediment was inoculated on LJ slant and incubated at 37°C for 8 weeks. Smears were prepared from each sediment and stained with ZN stain [17]. All positive LJ culture results were confirmed using MPT64 antigen method (Capilia TB) [18] and ZN smear microscopy.

### DNA extraction and drug susceptibility testing

The bacterial DNA from culture or decontaminated pellets were extracted using GenoLyse® DNA extraction kit. DNA extraction was performed as per the manufacturer’s instructions. Finally, the supernatant DNA solution was used for PCR or stored at -20°C until used. The master mix preparation, amplification, hybridization and detection steps were performed according to the manufacturer’s instruction. Molecular DST was performed using GenoType® MTBDRplus version 2 kit strips (to detect RIF and INH resistance) and GenoType® MTBDRsl version 2 kit strips (to detect FQs and aminoglycosides/peptide resistance) according to the manufacturer’s instructions (Hain Lifescience GmbH, Nehren, Germany). *M*. *tuberculosis* strain H37Rv (ATCC 27294) was used as sensitive control for each batch of DST procedure and known resistant strains were also used for each new kits.

### HIV testing

HIV test was done on whole blood using rapid HIV test algorithm of the Federal Ministry of Health of Ethiopia (KHB/STAT-PAK^®^/Unigold^™^ or Wantai/Uni-Gold/Vikia). The test was done at the Provider Initiative Counseling and Testing (PICT) services of the respective health institutions.

### Statistical analysis

Data were entered using Epi-Data version 3.1 and exported to SPSS version 20 (SPSS Inc., Chicago, Illinois, USA) for analysis. Data completeness and consistency was checked by running frequencies of each variable. Chi-square (χ2) or Fisher’s exact test, theoretical frequency less than five, was used to identify potential risk factors for development of drug resistant and p value < 0.05 was considered statistically significant. GPS (geographical positioning system) coordinates: longitude, latitude and altitude of each study participants and RR/MDR-TB treatment center hospitals location were collected and recorded using mobile GPS essential software (version 4.4.25 android) and Google Earth pro 7.3.2 software. Geo-locations were recorded on notebook and MS excel spreadsheet. The map location of DR-TB patients were constructed using ArcGIS 10.2.2 software to show the distribution of strains at district level.

### Ethical approval

The study protocol was approved by University of Gondar Ethical review board (Ref. No: O/V/P/RCS/05/19/2016) and permission letter was obtained from all study sites. Demographic and clinical data, and sputum samples were collected after obtaining written and/or oral informed consent from each study participants, and parent/guardian for those who were under age of 18 years old. Data confidentiality was maintained using secret codes for each participants.

## Results

### Demographic and clinical characteristics

A total of 216 pulmonary TB patients resistant to RIF based on GeneXpert RIF resistance detected or with history of treatment failure were included in the study. Of 216 patients, five were identified as non-tuberculosis mycobacteria (NTM) and were excluded from further analysis. The median age of the participants was 30 years (IQR: 24–42) and 63% were between 15–34 years age group. The majority of the study participants were males 63%, literate 62.1%) and urban dwellers 54%. Among the 211 participants, 64.5% were previously treated for TB disease, 25.6% were TB/HIV co-infected and 14.2% had contact history with RR/MDR-TB patient. One hundred twenty nine (61%) patients had positive sputum smear results. Eighty (38%) of the patients were admitted and followed their treatment at the University of Gondar referral hospital and 38.4% of the patients were living in North Gondar zone (Table 1).

**Table 1:** Demographic and clinical characteristics of study participants in the Amhara region (N = 211).

Characteristics	Category	n (%)
**Previous ant-tuberculosis treatment**	Yes	136 (64.5)
	No	75 (35.5)
**Previous contact with RR/MDR-TB patients**	Yes	30 (14.2)
	No	181 (85.8)
**Family history of tuberculosis**	Yes	35 (16.6)
	No	176 (83.4)
**HIV status**	Positive	54 (25.6)
	Negative	157 (74.4)
**Sex**	Male	133 (63)
	Female	78 (37)
**Age group, years**	15–24	58 (27.5)
	25–34	75 (35.5)
	35–44	33 (15.6)
	45–54	30 (14.2)
	≥55	15 (7.1)
**Residence**	Urban	114 (54)
	Rural	97 (46)
**Educational status**	Literate	131 (62.1)
	Illiterate	80 (37.9)
**Sputum ZN smear score**	Negative	82 (38.9)
	Scanty (1–9 AFB/100 fields)	12 (5.7)
	1+ (10–99 AFB/100 fields)	40 (19)
	2+ (1–10 AFB/field)	23 (10.9)
	3+ (>10 AFB/field)	54 (25.6)
**RR/MDR-TB treatment center Hospitals**	University of Gondar Hospital	80 (37.9)
	Boru Meda Hospital	34 (16.1)
	Woldia Hospital	22 (10.4)
	Ataye District Hospital	18 (8.5)
	Finote Selam Hospital	17 (8.1)
	Metemma Hospital	16 (7.6)
	Debre Bbirhan Hospital	9 (4.3)
	Debre Tabor Hospital	8 (3.8)
	Debre Markos Hospital	7 (3.3)
**Zonal location in the Amhara region**	North Gondar	81 (38.4)
	South Wollo	29 (13.7)
	North Shewa	24 (11.4)
	West Gojjam	21 (10)
	North Wollo	20 (9.5)
	South Gondar	11 (5.2)
	Agew Awi	9 (4.3)
	Oromia Special	7 (3.3)
	East Gojjam	5 (2.4)
	Wag Hemra	2 (0.9)
	Other region*	2 (0.9)

* Afar and Tigray region

RR: Rifampicin resistance; MDR-TB: Multi-drug resistant tuberculosis

### Resistance to INH and RIF

Among the 211 samples, 164 specimens were positive on LJ culture, 44 specimens yielded no growth and three specimens were contaminated. Of 211 isolates, 206 were RIF resistant by GeneXpert. Among 211 samples, 200 samples had interpretable results for first line drug resistance, and 11 were uninterpretable (6 invalid and 5 indeterminate) using line-probe assay kit and were excluded from the analysis. The DST result of 200 isolates demonstrated that 93% (186) isolates were RIF resistant and 7% (14) were susceptible using MTBDRplus assay (5 patients were treatment failure and 9 patients were RIF resistant by GeneXpert). In addition, 88.5% (177) and 11.5% (23) of isolates were resistant and susceptible to isoniazid, respectively. Overall, 87% (174) of isolates were confirmed as MDR-TB, 6% (12) were RIF mono-resistant, 2% (3) were INH mono-resistant and 6% (11) were susceptible for both RIF and INH using MTBDRplus assay. Of 174 MDR-TB patients, 63% (110) were previously treated with anti-TB drugs and 37% (64) were new cases. Thirty eight (22%) MDR-TB cases were HIV-positive.

### Resistance to second line drugs

Twenty six isolates were non-MDR-TB and were susceptible for second-line anti-TB drugs tested, and 174 isolates were MDR-TB strains. Among MDR-TB strains, 11 (6.3%; 95% CI: 2.9–10) were resistant to at least one second-line drugs. From 174 isolates, five (3.4%; 95% CI: 1–6) were resistant to FQs, 5 (3.4%; 95% CI: 1.1–6.2) were resistant to second line injectable drugs and one isolate was resistant for both FQs and second line injectable drugs. All of the isolates were susceptible for low level kanamycin. All MDR-TB patients had not been previously exposed to SLDs in the form of anti-TB therapy.

Among the 174 MDR-TB cases, the prevalence of pre-XDR TB was 10 (5.7%; 95% CI: 2.3–9.7) and XDR TB was 1 (0.6%; 95% CI: 0–2.3) at baseline before initiation of second line drug treatment. From 10 pre-XDR TB strains, 90% (9) were previously treated with anti-TB drugs, and one was from new cases. In addition, one XDR-TB was among new cases (Table 2). Among 11 strains with resistance to at least one SLD, 72% (8) isolates were found from patients in the age group of 15–34 years. More than half of resistant strains (6/11) were found from urban dwellers.

**Table 2:** Resistance to anti-TB drugs among new and previously treated RR/MDR-TB cases (N = 200).

Drug resistance pattern	New cases (n = 71) n (%)	Previously treated cases (n = 129) n (%)
Susceptible for both INH and RIF	5 (7)	6 (4.7)
RIF mono-resistance	1 (1.4)	11 (8.5)
INH Mono resistance	1 (1.4)	2 (1.6)
MDR-TB	62 (87.3)	101 (78.3)
MDR-TB + FQs	0	5 (3.9)
MDR-TB + KAN/CAP/VIO	0	1 (0.7)
MDR-TB + KAN/AMK/CAP	0	1 (0.7)
MDR-TB + AMK/KAN/CAP/VIO	1 (1.4)	2 (1.6)
MDR-TB + FQs + SLI	1 (1.4)	0

RIF: Rifampicin; INH: Isoniazid; MDR-TB: Multi-drug resistant tuberculosis; FQs: Fluoroquinolones; KAN: Kanamycin; AMK: Amikacin; CAP: Capreomycin; VIO: Viomaycin; SLI: Second line injectable drugs

### Distribution of drug resistant TB at district level

**Fig 2:**
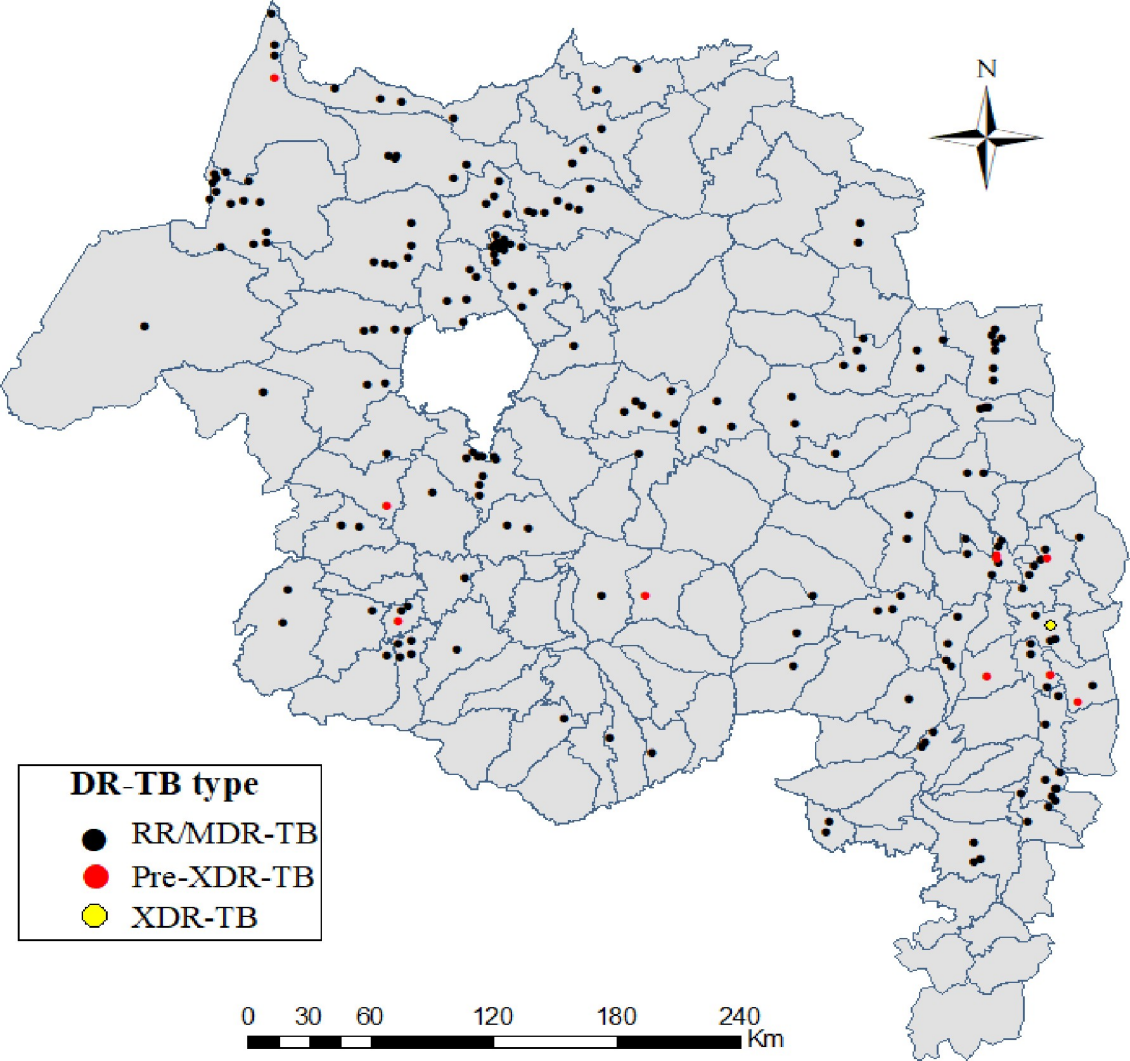
Distribution of drug resistant *M*. *tuberculosis* cases among the districts of Amhara region, Jan, 2016- Sept, 2018 (n = 209).

A total of 108 districts found in the Amhara region but DR-TB or treatment failed cases were occurred in 67 districts in the region during the study period (two cases were out of the region). The highest number of DR-TB cases were reported in districts of urban areas, Gondar (19 cases, 9%), Metemma (14 cases, 7%), Bahir Dar (9 cases, 4%) and Dessie (8 cases, 4%). Two Pre-XDR-TB cases were found in Dessie and the others were distributed in eight districts, and XDR-TB was found in Dawa Harewa district, Oromia special zone. The map shows the distribution of drug resistant TB cases in the Amhara region at district level (Fig 2).

## Discussions

Early detection of SLDs resistance have a key importance to optimize treatment regimen composition and to direct infection control measures to block transmission of RR/MDR-TB. The high levels of second-line resistance are major causes of concern globally, especially in resource limited countries like Ethiopia and have important implications for the use of short treatment regimens, new therapeutic agents and introduction of new rapid diagnostic tools.

All study participants were initiated treatment with SLD regimen based on WHO and Ethiopia National protocol. The WHO defines GeneXpert RIF resistance as a prognostic marker for MDR-TB. From 14 RIF susceptible strains, 5 were obtained from treatment failure and 9 were RIF resistant by GeneXpert but susceptible by MTBDRplus assay. Our study was supported by other study that GeneXpert MTB/RIF assay incorrectly assigned RIF resistance in 31% of cases and MDR-TB may be over-diagnosed resulting suboptimal treatment regimens [19]. But, this discordant result needs to be checked by the gold standard phenotypic DST methods in order to know which molecular test is incorrect.

In addition, FLD treatment failure patients were initiated for SLD treatment due to resistant to the most potent anti-TB drug, INH although they were susceptible to rifampicin. The clinicians should select appropriate anti-TB treatment regimen via DST at baseline, especially use of high dose INH for treatment of INH susceptible RR-TB strains to improve the management and treatment outcome of patients. Furthermore, improved infection prevention and control strategy is mandatory in RR/MDR-TB treatment centers/ facilities during treatment follow up as healthcare facilities could serve as the sites for the spread of drug resistant TB [20].

The rate of pre-XDR and XDR-TB in our study were 5.7% and 0.6%, respectively. Compared to our findings, majority of studies reported higher rate of pre-XDR TB in India (56%), China (34%), Pakistan (24%), Bangladesh (16%), Nigeria (17%) and South Africa (17%) [21–25]. The high rate of pre-XDRTB in China, India and other countries compared to our study might be connected to the fact that China and India have the highest rate of MDR-TB in the world and a better laboratory setup and resources for the isolation of drug resistant strains. Identification of pre-XDR TB patients will assist clinicians to monitor these patients closely and prevent the progression to XDR-TB which is more difficult to treat and poor treatment success.

Moreover, our finding of XDR-TB is similar with the study conducted in Ethiopia (1%), Pakistan (2%), Thailand (0.38%) and Iran (0.2%) [26–28]. Higher rate of XDR-TB were reported among MDR-TB patients in multi-center study in eight countries (6.7%), Estonia (5.2%), India (11%), Vietnam (6%), India (4.85%), Poland (6.4%) and Addis Ababa (4.4%) [21, 29–34]. Acquired SLD resistance was defined as any SLD whose status changed from susceptible at baseline to resistant at follow up [35]. This could be due to the fact that most of the study areas are considered as the highest burden of TB/MDR-TB in addition to the variation on the study area population, geographical location and time of sputum samples collection which can be collected after initiation of second line drugs. An MDR-TB cohort study in Georgia on DST of SLDs at baseline and every third month showed that 14% (9.1% for FQs, 9.8% for KAN/CAP and 9.9% for XDR-TB) of follow up positive cultures for *M*. *tuberculosis* isolates have acquired drug resistance [35].

Based on our observation in the Amhara region, some patients with MDR-TB initially susceptible to SLDs at baseline were found to be resistant to at least one SLD after 3 months of MDR-TB treatment initiation but this result was not included in our study.

Among 10 pre-XDR-TB isolates, 90% were previously treated with first line drug for active TB disease although not statistically significant (p = 0.07). This variation in drug resistance patterns of MDR-TB isolates is due to mutational heterogeneity in mycobacterial genes associated with anti-TB drug resistance [36], and also could be due to transmission from person-to-person and de novo evolution of resistance. WHO suggested that treatment decisions should be guided based on the patient’s clinical history and recent surveillance data in settings with lack of SLDs DST [37].

The incidence of FQs resistance was 3.4% (6/174) in our study. This finding is similar with other studies reported in Delhi (7.5%), Pakistan (7%) and Zimbabwe (2%) [38–40]. The incidence of second line injectable drugs resistance was 3.4%. Several other studies have found much higher rate of FQs resistance in MDR-TB patients, reported as 10%, 13%, 24%, 35% and 57% [41–45]. The increase in FQ resistance might be due to extensive prescription of FQs for treatment of respiratory and other bacterial infections and easy accessibility in some areas even available in local drug markets without prescription. A strict regulatory policy for FQs dispensing by prescription only would decrease misuse of the drug and decrease the emergence of DR-TB.

In our study, approximately 65% of MDR-TB cases were previously treated. One XDR-TB patient was among the new MDR-TB cases. This finding might indicate a significant public health threat given that there could be a progressive drug resistant strain transmission in the population. Therefore, this finding could be an important indicator for the health care system in the Amhara region and in Ethiopia to design and implement a more effective TB treatment, diagnostic, and prevention and control programs.

The present study found a higher number of RR/MDR-TB (63%) cases among 15–34 years of age group. Moreover, 70% (7/10) of pre-XDR-TB cases were among 15–34 years of age group (p = 0.318). This finding is consistent with other studies done in India and Nigeria that reported higher rate of pre-XDR-TB cases among the young adult groups, with age groups 18–25 years and 15–29 years, respectively [21, 22]. This higher rate of DR-TB in the active age groups might be due to frequent movement, increased outdoor contact and higher case notification due to greater health awareness among young adults.

Furthermore, more than half of the RR/MDR-TB and also pre-XDR/XDR-TB cases lived in urban settings especially in Gondar, Metemma and Bahir Dar cities. This finding is supported by previous report in central and southwest Ethiopia that patients who live in urban area are more likely to have DR-TB due to overcrowding/ slum areas that favor transmission of TB [46, 47] and also may be due to urbanization, high inequalities to health service access and quality of care (homeless, migrants, alcohol abusers and illegal drug users) and ecological factors. Previous study supports that high temperature and low altitude areas increases the TB incidence [48, 49].

We found that DR-TB was disproportionately distributed in districts of the Amhara region. At zonal level, 39% of cases were found in north Gondar zone which borders with Tigray region and Sudan. The possible explanation for this could be that there were frequent cross-border population movements from the neighboring border areas Tigray region and Sudan for economic and social reasons, which could favor the disease transmission in these areas. Transmission in border areas was supported by other study in Poland [50], and establishing a cross-border collaboration network might be help to reduce the disease burden in these areas. The control of DR-TB depends on early diagnosis of resistance, appropriate therapy, improve patient's awareness for TB treatment, and interrupting transmission chains. Therefore, future TB prevention, care and management efforts in these areas should focus on strengthening health care infrastructure, increase laboratory capacity for early diagnosis and treatment, monitoring of individuals with TB symptoms and for baseline SLDs DST. Utilization of DOTS and DOTS-Plus strategy in the use of SLDs and increasing community awareness are also crucial.

## Conclusions

In conclusion, the rate of at least one second line drug resistance among MDR-TB patients is alarming in the region. Conducting DST at the baseline is recommended for RR/MDR-TB patients to monitor and evaluate the pre-XDR and XDR-TB patient status during the treatment course to ensure clinical improvement and adjust treatment regimen to prevent treatment failure and also reduce its transmission to the community in the study area and the country at large. Further study is warranted to explore the role of various ecological factors on the observed high distribution of DR-TB and identify transmission network in the community using molecular epidemiological methods to locate hotspots for targeted interventions.
